# Diet and physical activity intervention in colorectal cancer survivors: A feasibility study

**DOI:** 10.1016/j.ejon.2014.08.006

**Published:** 2015-02

**Authors:** Chloe Grimmett, Alice Simon, Victoria Lawson, Jane Wardle

**Affiliations:** Health Behaviour Research Centre, Department of Epidemiology and Public Health, University College London, Gower Street, London, WC1E 6BT, UK

**Keywords:** Cancer survivors, Intervention, Physical activity, Diet, Quality of life, PA, Physical activity, F&V, Fruit and vegetables, CRC, Colorectal cancer, SES, Socioeconomic status, MVPA, Moderate and vigorous physical activity, MID, Minimally important differences

## Abstract

**Purpose:**

Evidence that lifestyle factors are associated with better outcomes in colorectal cancer (CRC) survivors highlights the need for behaviour change interventions. This study examined feasibility and acceptability, and provided an indication of behavioural impact, of a telephone-based, multimodal health behaviour intervention for CRC survivors.

**Method:**

Participants were recruited from five London hospitals. Patients (*n* = 29) who had recently completed treatment for CRC participated in a 12 week intervention. Behavioural goals were to increase physical activity (PA) and fruit and vegetable (F&V) intake, and reduce consumption of red/processed meat and alcohol. Self-report measures of PA and diet were completed in all patients, supplemented by objective measures in a sub-set.

**Results:**

Uptake of the study when patients were approached by a researcher was high (72%), compared with 27% contacted by letter. Methods for identifying eligible patients were not optimal. Study completion rate was high (79%), and completers evaluated the intervention favourably. Significant improvements were observed in objectively-measured activity (+70 min/week; *p* = .004). Gains were seen in diet: +3 F&V portions a day (*p* < .001), −147 g of red meat a week (*p* = .013), −0.83 portions of processed meat a week (*p* = .002). Changes in serum vitamin levels were not statistically significant, but the small sample size provides limited power. Clinically meaningful improvement in quality of life (*p* < .001) was observed.

**Conclusion:**

An intervention combining print materials and telephone consultations was feasible and acceptable, and associated with improvements in PA, diet and quality of life.

## Introduction

Epidemiological studies have consistently shown that diet (Martinez, 2005) and physical activity (PA) (Wolin et al., 2009) are associated with the risk of developing colorectal cancer (CRC). Evidence is also emerging that these factors are associated with survival after a diagnosis of CRC: all-cause and cancer mortality were lower among active than inactive CRC survivors (Meyerhardt et al., 2006a, 2006b), and rates of CRC recurrence and mortality were lower in those consuming a ‘prudent’ than a ‘western’ diet (Meyerhardt et al., 2007). Healthy behaviours among cancer survivors have also been associated with better quality of life, reduced fatigue, and improved physical function (Doyle et al., 2006).

In the light of evidence that healthy lifestyle promotes survival, cancer survivors are now recommended to adhere to population guidelines for cancer prevention; namely maintain a healthy weight, engage in regular physical activity, eat at least five portions of fruit and vegetables (F&V) a day, limit alcohol, limit processed and red meat consumption, and not smoke (World Cancer Research Fund & American Institute of Cancer, 2012). However, studies across several countries find little evidence that cancer survivors as a group are adopting healthier lifestyles. A population-based survey in the UK found lower levels of PA among cancer survivors than the general population (Grimmett et al., 2009) and surveys in Australia and the US showed that cancer survivors were overweight, consumed insufficient F&V and fibre, and were inactive (Blanchard et al., 2008; Coups and Ostroff, 2005; Eakin et al., 2007). CRC survivors have been found to have the lowest physical activity rates of any cancer group (Courneya et al., 2008).

There is therefore a need to identify effective and acceptable interventions for behaviour change in colorectal cancer survivors. A feasibility study of an intensive lifestyle intervention (involving multiple home visits from a counsellor) in overweight CRC survivors found high adherence and modest behaviour change (Anderson et al., 2010). Two other studies focused specifically on PA (e.g. Bourke et al., 2011; e.g. Pinto et al., 2005). Multiple behaviour change interventions among survivors of other cancers using personalised and tailored print materials have given promising results (Demark-Wahnefried et al., 2007). Telephone counselling (Pinto et al., 2005; Morey et al., 2009) is potentially a lower cost delivery method that obviates the need for travel and time commitments.

The present study examined the feasibility and acceptability of a telephone-based intervention with print materials targeting multiple behaviour change in patients who had recently completed treatment for CRC. Behavioural targets were in line with population recommendations: 150 min of physical activity a week, five or more portions of F&V a day, limited alcohol consumption, <500 g of red meat a week, and no processed meat. The intervention was delivered in two phases varying only in the method of recruitment and the inclusion of objective measures of behaviour. Phase One used face-to-face recruitment and predominantly self-report measures of behaviour. In Phase Two, recruitment was carried out by mail or using research nurses, and objective measures of behaviour were added to the assessment protocol. Data from the two phases were combined for estimates of acceptability and self-reported behaviour change, while objective measures of behaviour change were available only in Phase Two.

## Methods

### Population

Inclusion criteria were having completed treatment for non-metastatic CRC within the last 6 months, being over 18 years old, and, because translation was not affordable for the pilot study, to have reasonable spoken and written English. Potential participants were included if they had adequate mobility and no contraindications for unsupervised physical activity (e.g. without major health problems or sub-total or total colectomy or ileostomy).

### Recruitment

*Phase One*: Phase One was conducted as part of a PhD study. Patients were recruited from three London hospitals. At one site the researcher (CG) attended weekly clinics. Eligible patients were given information by the consultant and referred to CG for further information. At the two other sites, the same consultant identified eligible patients and gave information about the study. They were then given a letter of invitation and study information, and invited to call the research office or return the reply slip.

*Phase Two*: Phase Two was an extension of the PhD work, designed to explore alternative recruitment methods and include objective measures of behaviour change. Research nurses were responsible for recruitment across five London hospitals. In the first instance patients who had been discharged from curative treatment within the past six months were identified by the nursing team and contacted by post. From that point on, patients attending end of treatment appointments were identified and the research nurse informed them about the study. Patients were posted/given a letter of invitation signed by the consultant, information sheet, and postage paid reply slip.

### The intervention

The intervention was designed to encourage increases in daily PA and F&V intake and reductions in red and processed meat intake to meet recommended population guidelines (150 min of PA a week, five servings of F&V a day, <500 g of red meat a week, no processed meat). In Phase Two, patients were additionally encouraged to consume alcohol within recommended limits (no more than 21 units a week for men and 14 units for women). At the start of the intervention, they were sent written information describing evidence for the benefits of healthy lifestyle for CRC survivors. Examples of seated and standing exercises that could be done in the home were provided, along with information on portion sizes, examples of meat-free menus, and work-books to use throughout the study.

The intervention lasted 12 weeks, with two-weekly telephone consultations from CG. Consultations were guided by self-regulation theory in accordance with a recent meta-analysis which found interventions using self-regulation theory to promote healthy eating and PA were more effective (Michie et al., 2009). Behaviour change techniques included specific goal-setting, review of behavioural goals, self-monitoring of behaviour, and feedback on performance. Social support was encouraged. Patients made their own choice of whether to begin with PA or diet changes. Once improvements in the first behaviour were achieved, changes in the second behaviour were introduced.

### Assessment

Patients attended a baseline assessment at the university. Informed consent was taken, questionnaire measures of diet and psychosocial variables were completed, and height and weight were measured. Patients in Phase One recorded their daily pedometer step count for three days before the intervention. For Phase Two, accelerometers were worn for seven days before the intervention to generate both step counts and time spent in PA. Blood samples were taken for vitamin assays of ascorbic acid (Vitamin C), plasma alpha-tocopherol (Vitamin E) and plasma beta-carotene. On completion of the intervention, patients attended a follow-up assessment using the same assessment protocol. All those in Phase One and a sub-sample from Phase Two completed telephone interviews to explore intervention acceptability.

### Measures

*Demographics*. Participants were asked to report age, sex and marital status. Socioeconomic status (SES) was indexed using a combination of material circumstances and education; car ownership vs. not, home ownership vs. not and university-level education vs. not. Summing these items generates a score between 0 (no deprivation) and 3 (high deprivation). For analysis this score was dichotomised into 0 vs. ≥1. This measure was used as the majority of participants were retired and therefore occupation and income are not as reflective of SES as in younger adults (Wardle et al., 1999).

*Physical activity*. Self-reported PA was measured using the modified version of the Godin Leisure Time and Exercise Questionnaire (Godin et al., 1986) which asks about frequency and average duration of bouts of mild, moderate and vigorous activity in the last seven days. Total time spent in moderate and vigorous activity (MVPA) a week was calculated. In Phase One, step counts a day were measured using a pedometer (Yamex digiwalker SW-200), with patients recording total number of steps for three days, from which average daily step count was calculated. In Phase Two, activity, including step counts, was assessed using accelerometry. Patients were asked to attach the accelerometer when they woke and to remove it when they went to bed. Activity was monitored in one minute epochs, from which time spent in moderate (2000–3999 counts a minute; cpm) and vigorous (≥4000 cpm) activity in at least 10 min bouts were calculated. These cut-offs have been used in samples of older adults (Davis and Fox, 2007; Harris et al., 2010).

*Diet*. Consumption of F&V, red and processed meat was assessed using a modified version of the Health Education Authority (HEA3) food frequency questionnaire (Little et al., 1998) Patients were asked to estimate portion sizes (small, medium, large), the number of days each week the food was consumed, and the number of portions a day. Red meat was calculated in grams a week, processed meat in portions a week, and F&V in portions a day. As an objective measure of F&V consumption, plasma ascorbic acid (Vitamin C), plasma alpha-tocopherol (Vitamin E), and plasma beta-carotene were measured in Phase Two (Cappuccio et al., 2003). Blood was drawn into a light protected tube and samples were left to clot for 30 min, centrifuged, and the serum transferred into separate light-protected vials. Analysis involved high-performance liquid chromatography and single wavelength ultraviolet detection.

*Quality of life, fatigue and physical function*. Quality of life was assessed using the Functional Assessment in Cancer Therapy-Colorectal (FACT-C) scale; a 36 item questionnaire in which higher scores indicate better QoL. Total scores (range 0–136) and subscales of physical, functional, social and emotional wellbeing, as well as a colorectal-specific scale, were calculated. The minimally important difference (MID) is 5–8 points for the FACT-C total score (Yost et al., 2005). Fatigue was measured using the Functional Assessment of Cancer Therapy-Fatigue (FACT-F) scale (Yellen et al., 1997); a 13-item scale with higher scores indicating greater fatigue and an MID of 3 points (Cella et al., 2002). The physical function subscale of the SF-36 (v2) was used to measure functional status. This validated 10-item questionnaire has a score range from 0 to 100, with higher scores indicating better physical function (Cella et al., 2002).

*Acceptability*. Adherence to telephone counselling sessions and attendance at follow-up were used as one indicator of acceptability. Interviews were used to explore the acceptability of the length and format of the intervention, the timing of delivery (in relation to cancer diagnosis and treatment) and overall perceived usefulness.

### Analysis

Descriptive statistics were used for patient characteristics and for diet, activity and QoL at baseline and follow-up. Paired *t*-tests and chi square were used to examine the statistical significance of changes from baseline to follow-up. Where the same measures were used across Phases One and Two, results were combined (*N* = 29).

## Results

The average age of the total sample (*N* = 29) was 65 years, ranging from 44 to 79 years. Just over half the patients (62%), were female, 50% reported no markers of deprivation, 24 (78%) were white and 62% (*N* = 18) were married. The average BMI was 26 kg/m^2^. Those in Phase One were slightly older than those in Phase Two (66.5 and 63.4 years, *p* = .020), but there were no other significant differences.

### Recruitment

*Phase One*: Over a four month period, 18 patients were identified who met the inclusion criteria and 13 (72%) were interested in taking part. One was unwell and could not attend the baseline assessment and subsequently withdrew, another was already meeting all behavioural targets. The final sample size was 11.

Uptake from those approached in Phase One was high. However due to limited resources identification of potentially eligible patients was not optimal. Clinicians estimated 40 patients meeting the inclusion criteria per site per year; a possible 67 patients over the four month recruitment period, compared to the 18 identified and approached.

*Phase Two*: 57 patients were invited by post to take part in the study and 15 (26%) responded to say they would like to take part, of whom 13 were eligible. A further five were recruited by research nurses on discharge of curative treatment. Recruitment rates by research nurses could not be calculated as they were not able to meet our request to record data on the number of patients approached to take part. Based on estimates as per Phase One, as many as 133 patients may have been eligible over the recruitment period. The final sample size was 18.

### Attrition, compliance and adverse events

One patient from Phase One withdrew midway through the study citing personal problems (attrition rate 9%). Five withdrew from Phase Two; two with a recurrence and three because of loss of interest or time constraints (attrition rate 28%). A total of 23 patients completed the trial and are included in the final analyses.

Of the 23 patients who completed the trial, 18 completed all scheduled telephone consultations and five each missed no more than one consultation (96% compliance).

One digestive upset was reported and was attributed by the patient to eating more F&V.

### Behaviour change

Self-report measures of PA (Phases One and Two) showed significant increases in both moderate and vigorous activity (Table 1); with 52% achieving the recommended ≥150 min a week at the end of treatment, compared with 13% at baseline. By the end of treatment, 23% were meeting the behavioural target of 10,000 steps a day compared with 9% at the start of the study. More conservative improvements were seen with accelerometry (Table 2), but results still showed an increase of 70 min of activity a week (*p* = .007), and 52% of patients met the recommended levels at follow-up compared with 13% at baseline.

Self-reported F&V consumption increased significantly (*p* < .001), with mean intake exceeding 7 portions a day at follow-up. All patients were consuming at least 5 portions a day, compared with only 30% at baseline. Serum vitamin assays were used to assess the feasibility of objective measures in Phase Two. The largest change was in Vitamin C, which increased by 5 μmol/L (Table 2). However, none of the changes reached significance in this small sample (*N* = 12).

Red meat intake was significantly reduced (*p* = .013), with only one person consuming more than 500 g a week. Consumption of processed meat reduced, with 78% consuming no processed meat at follow-up compared with 35% at baseline (Table 1).

Alcohol consumption was generally low. However one man was a heavy drinker and his results suggested an increase from 70 units a week at baseline to 110 units a week at follow-up. Average consumption at baseline (excluding this individual) was 7 (sd = 10.8) units a week falling to 4 (sd = 9.1) units a week at follow-up. Only two others (both female) exceeded recommended guidelines of alcohol consumption at baseline; of whom one reduced her intake from 15 units a week to no alcohol at follow-up, but the other made little change, consuming 34 units at baseline and 32 at follow-up.

### Psychosocial outcomes

Table 3 presents changes in psychosocial outcomes. A clinically significant increase of 7 points was observed in total QoL, as well as notable increases in functional well-being (+4; *p* = .077). All other scores changed in the hypothesised direction, although they did not reach statistical significance.

### Patient acceptability

Telephone consultations as a method of intervention delivery were positively received, with several patients remarking on the convenience of not travelling for appointments. Nonetheless, they valued the face-to-face meeting during baseline assessment as an opportunity to build rapport with the researcher: “*going out for that first meeting helped the telephone because you had a face to face there … so I think that made the calls that bit more personal*”. The timing of the intervention in relation to completion of cancer treatment was deemed appropriate: “*I think there's a window there, while people are still interested in what they've been through, the surgery and all that, and to keep it rolling*”. The intervention was also generally perceived as a helpful and useful exercise: “*We [participant and wife] did enjoy it, yes, no doubt about that. And it opened our eyes to diet and exercise, which if we hadn't come we wouldn't know about*”.

## Discussion

The primary aim of this study was to determine if the intervention was acceptable and feasible. The results gave strong support on both counts. Attrition rates were low, with only four participants (14%) withdrawing from the study, and compliance with the telephone consultations was high (96%). End of treatment interviews showed that patients rated the intervention favourably.

Self-reported PA increased significantly and there was an average increase of almost 1300 steps a day. Accelerometry in Phase Two showed an average increase of 70 min of activity a week, with 52% participants meeting the recommended 150 min a week. The differences between self-report and accelerometry is likely to be due to self-report bias, and there is general agreement that self-reports over-estimate activity (Troiano et al., 2008).

There were significant increases in self-reported F&V consumption from baseline to follow-up, with all participants reporting at least 5 portions of F&V a day. The data from the vitamin assays did not show significant increases, although vitamin C changed in the hypothesised direction, thus we did not confirm the self-report results in this small sample. The intervention was also associated with significant changes in self-reported meat consumption, with all but one patient meeting the recommendation of <500 g of red meat a week, and 78% reporting no processed meat consumption at follow-up.

Alcohol consumption was only assessed in Phase Two and was low at baseline. This is consistent with results from a UK population-based study which found that only 8% of respondents consumed two or more drinks a day (Grimmett et al., 2009). Results from the one very heavy drinker suggested an increase in consumption at follow-up. However, our clinical impression was that he had significantly underestimated his intake at baseline and actually reduced his intake over the course of the study (this was also his view). It appears that interventions to reduce alcohol intake may only be applicable to a sub-group of this population and might need further attention.

The extent of behaviour change in this study compares favourably with findings in the literature. A similar study with overweight CRC survivors (Anderson et al., 2010) reported comparable increases in PA (72 min a week) and improvements in F&V consumption as reflected by increases in ascorbic acid.

Other encouraging findings included clinically significant improvements in total QoL, as well as improvements in functional well-being. This is consistent with accumulating evidence for better QoL and physical function in cancer survivors who adhere to healthful lifestyles (Blanchard et al., 2008; Grimmett et al., 2011; Mosher et al., 2009).

One outcome of interest was the effectiveness of different recruitment strategies. Phase One was part of a PhD study, with one researcher (CG) and one consultant responsible for identifying and approaching eligible patients. With such limited resources only 18 of an estimated 67 eligible patients (40 per site per year) were approached. However uptake among those approached was excellent (72%). Approaching patients by letter in Phase Two was less effective (26%), although this compares favourably with previous studies using similar recruitment strategies. In one recent study, uptake to a lifestyle intervention was only 10% (Bourke et al., 2011). During Phase Two, in addition to approaching patients by letter, research nurses were responsible for identifying patients and informing them about the study. Recruitment by this method was very disappointing, with only five patients successfully recruited despite the involvement of five hospitals over an 8 month period. Furthermore, nurses were not able to comply with the request to keep data on patients approached, due to being over-stretched (personal communication) and having competing responsibilities. The successful recruitment in Phase One was likely due to a motivated researcher having sufficient time to discuss the trial fully with each patient after endorsement of the trial by their physician. If recruitment by research nurses in future trials is to be maximised, motivation to recruit would need to be enhanced, and prioritisation of lifestyle studies at participating sites ensured.

The interpretation of these results is inevitably limited by the lack of control group which makes it impossible to be certain that the observed changes were due to the intervention. The sample size was also small, especially for the objective measures, limiting statistical power, but we were able to indicate that collecting the objective data posed no difficulty. The 75% recruitment rate in Phase One suggests that the level of enthusiasm for lifestyle advice in CRC survivors is high, but other methods of recruitment fell far short. Excluding patients with sub-total or total-ileostomy limits the generalisability to all CRC survivors, as does the high proportion of female participants, with relatively low BMI and high SES.

In summary, this study of a multiple health behaviour intervention is one of few to use objective measures of physical activity to assess the impact of a lifestyle intervention in CRC survivors and target consumption of red and processed meats as part of the dietary intervention. Patient interest was high when approached directly and acceptable with mailed information. Positive changes in physical activity were seen with both self-report and objective measures, and for diet using self-report. The relatively high uptake, good compliance, positive effects on health behaviours and quality of life, and efficiency of the delivery system, make a case for a full-scale randomised controlled trial.

## Conflict of interest

None to declare.

## Figures and Tables

**Table 1:** Self-reported behaviour change.

	T0	T1	Mean change	*T*-test/χ^2^
				*p*
Physical activity (minutes a week)				
aModerate (*N* = 22) *Missing* = *1*	48.3 (73.2)	123.5 (92.0)	+73.0 (115.4)	*T* (21) = 2.96, *p* = .007
bVigorous (*N* = 23)	14.78 (35.4)	67.17 (113.5)	+52.4 (116.6)	*T* (22) = 2.16, *p* = .042
Physically active >150 min a week (*n* %)	3 (13%)	12 (52%)	–	χ^2^ = .63, *p* = .427
F&V (portions a day) *N* = 23	4.2 (2.0)	7.1 (1.7)	+2.89 (1.77)	*T* (22) = −7.78, *p* < .001
≥5 portions a day (*n* %)	7 (30%)	23 (100%)	–	–
Red meat g a week *N* = 23	262.7 (310.5)	115.3 (132.8)	−147.4 (263.1)	*T* (22) = 2.69, *p* = .013
<500 g a week (*n* %)	18 (78%)	22 (96%)	–	χ^2^ = 3.764, *p* = .052
Processed meat (portions a week) *N* = 23	1.17 (1.15)	0.35 (0.78)	−0.83 (1.15)	*T* (22) = 3.43, *p* = .002
Consuming no processed meat (*n* %) *N* = 23	8 (35%)	18 (78%)	–	χ^2^ = 3.407, *p* = .065

a Moderate physical activity = when you breath somewhat harder than normal e.g. brisk walking.

b Vigorous physical activity = when your heart beats rapidly and you breath much faster e.g. running.

**Table 2:** Objective measures of behaviour change.

	T0	T1	Mean change	*T*-test
				*p*
Step counts per day *N* = 22 *Missing* = *1*	5769 (3035)	7293 (2937)	1299 (1508)	*T* (21) = 4.04, *p* = .001
≥10,000 steps per day (*n* %)	2 (9%)	5 (23%)	–	χ (1) = 7.48, *p* = .006
aPA (bouts >10 min) *N* = 13	97 (105)	168 (129)	70 (71)	*T* (12) = 3.52; *p* = .004
Physical active >150 min a week (*n* %)	2 (15%)	7 (54%)	–	χ (1) = 2.03, *p* = .115
IGF-1 μmol/L (*N* = 13)	18.3 (4.8)	16.4 (4.1)	−1.1	*T* (11) = 1.02, *p* = .332
Ascorbic acid μmol/L (*N* = 13)	46.6 (26.6)	51.6 (23.5)	+5.1 (21.7)	*T* (12) = .838, *p* = .481
Beta carotene μmol/L (*N* = 13)	1.4 (1.6)	1.2 (0.7)	−0.28	*T* (11) = .679, *p* = .511
Alpha-tocopherol μmol/L (*N* = 13)	90.0 (24.2)	87.7 (23.0)	−2.24	*T* (12) = .416, *p* = .685
BMI (*N* = 22) *Missing* = *1*	26.4 (4.3)	26.6 (4.3)	0.08	*T* (21) = .531, *p* = .601

a PA = Total of moderate (2000–3999 cpm) + vigorous activity (≥4000 cpm).

**Table 3:** Psychosocial measures.

*N* = 23	T0	T1	Mean change	*T*-test
				*p*
Quality of life				
Total score (0–136)	91.5 (11.1)	98.5 (13.4)	7.01 (13.6)	*T* (22) = 2.518, *p* = .020
PWB	24.7 (2.9)	25.6 (2.7)	0.93 (2.8)	*T* (22) = 1.56, *p* = .130
EWB	20.7 (2.8)	21.6 (2.1)	0.90 (3.1)	*T* (22) = 1.42, *p* = .170
FWB	23.3 (4.1)	27.3 (2.9)	4.01 (10.4)	*T* (22) = 1.86, *p* = .077
SWB	22.8 (6.9)	24.0 (5.1)	1.18 (7.4)	*T* (22) = .763, *p* = .454
CCS	23.4 (2.5)	23.9 (2.7)	0.50 (2.0)	*T* (22) = 1.20, *p* = .242
Fatigue	7.5 (6.5)	6.9 (7.7)	−0.9 (7.9)	*T* (22) = 5.25, *p* = .605
SF-36 (physical function)	81.7 (15.5)	85.0 (16.2)	3.4 (17.3)	*T* (21) = .926, *p* = .365

PWB = physical well being, FWB = functional well being, SWB = social well being, EWB = emotional well being, CCS = colorectal cancer scale. Higher scores indicate better QoL.
